# Long-Term Evolution of Brainstem Electrical Evoked Responses to Sound after Restricted Ablation of the Auditory Cortex

**DOI:** 10.1371/journal.pone.0073585

**Published:** 2013-09-16

**Authors:** Verónica Lamas, Juan C. Alvarado, Juan Carro, Miguel A. Merchán

**Affiliations:** 1 Instituto de Neurociencias de Castilla y León, Universidad de Salamanca, Salamanca, Spain; 2 Instituto de Investigación en Discapacidades Neurológicas (IDINE), Facultad de Medicina de Albacete, Universidad de castilla-La Mancha, Campus in Albacete, Albacete, Spain; University of Fukui, Faculty of Medical Sciences, Japan

## Abstract

**Introduction:**

This study aimed to assess the top-down control of sound processing in the auditory brainstem of rats. Short latency evoked responses were analyzed after unilateral or bilateral ablation of auditory cortex. This experimental paradigm was also used towards analyzing the long-term evolution of post-lesion plasticity in the auditory system and its ability to self-repair.

**Method:**

Auditory cortex lesions were performed in rats by stereotactically guided fine-needle aspiration of the cerebrocortical surface. Auditory Brainstem Responses (ABR) were recorded at post-surgery day (PSD) 1, 7, 15 and 30. Recordings were performed under closed-field conditions, using click trains at different sound intensity levels, followed by statistical analysis of threshold values and ABR amplitude and latency variables. Subsequently, brains were sectioned and immunostained for GAD and parvalbumin to assess the location and extent of lesions accurately.

**Results:**

Alterations in ABR variables depended on the type of lesion and post-surgery time of ABR recordings. Accordingly, bilateral ablations caused a statistically significant increase in thresholds at PSD1 and 7 and a decrease in waves amplitudes at PSD1 that recover at PSD7. No effects on latency were noted at PSD1 and 7, whilst recordings at PSD15 and 30 showed statistically significant decreases in latency. Conversely, unilateral ablations had no effect on auditory thresholds or latencies, while wave amplitudes only decreased at PSD1 strictly in the ipsilateral ear.

**Conclusion:**

Post-lesion plasticity in the auditory system acts in two time periods: short-term period of decreased sound sensitivity (until PSD7), most likely resulting from axonal degeneration; and a long-term period (up to PSD7), with changes in latency responses and recovery of thresholds and amplitudes values. The cerebral cortex may have a net positive gain on the auditory pathway response to sound.

## Introduction

Rasmussen's early anatomical studies showed the existence of descending connections along almost all relay stations in the auditory pathway [1]. The end effectors of this chain are the olivo-cochlear neurons, which in turn regulate the inner ear responses. This efferent pathway feedback on cochlear responses was masterfully shown in electrophysiological experiments performed by Robert Galambos, who found that electrical stimulation of olivo-cochlear fibres causes a drop in the electrically evoked activation of the cochlea after sound stimulation [2].

Recent anatomical studies have shown direct cortical projections to cochlear nuclei and the olivary complex, which includes the ventral nucleus of the trapezoid body (VNTB), lateral superior olive (LSO) and dorsal periolivary region of the LSO [3], [4], [5]. The combination of retrograde and anterograde tracing has shown close contacts between medial olivo-cochlear (MOC) neurons and corticofugal terminals in the VNTB [4]. Anterograde tracers injected in the temporal cortex also showed terminal fields from the auditory cortex (AC) in the LSO and dorsal periolivary region [3], wherein lateral olivo-cochlear (LOC) neurons are located. These findings and the presumably excitatory character of the corticofugal pathway [6] suggest that the cerebral cortex may activate both MOC and LOC neurons and its efferent circuits to the cochlea and cochlear nuclei.

The physiological effects of the descending control on the responses of the olivo-cochlear efferent system have been analyzed in experiments of electrical stimulation, chemical blockade, cooling or ablation of the AC. Electrical stimulation of AC has shown changes in the inner ear cochlear microphonic potentials [7] and a decrease in amplitude of the otoacoustic emisions [8]. An increase in response magnitude and shortening in the response latency of cochlear nuclei has also been shown after AC electrical stimulation [9], [10]. The chemical or physical blockage (cooling) of the AC has also enabled to analyze its effects on the peripheral processing of sound [7], [11]. A cortical ablation animal model for analyzing the descending control effect on auditory startle reflex and auditory brainstem responses (ABR) has been previously used by Hunter and Willot [12]. These authors observed minimal changes in ABR threshold, no changes in latencies but a decrease in ABR amplitudes 1 day after ablation and recovery 30 days later.

A different ipsi- versus contralateral effect of cortical deprivation (AC ablation) may be expected on electrically evoked activity in the brainstem because the descending projection to the brainstem ends bilaterally, albeit with a weak contralateral component [3], [5], [13].

This study aimed to shed light on the effect of top-down control on the lower auditory pathway by comparing ABRs assessed after unilateral vs. bilateral cortical lesions. Previous findings from our laboratory have shown reversible changes in c-Fos protein expression, calretinin immunostaining and gene expression in the midbrain after AC ablations, suggesting the long-term regulation of post-lesion plasticity in the auditory pathway [14], [15], [16].

This study was conducted to gain insight on the role of the descending control in hearing and aimed to examine the long-term self-repair compensation of the AC at different post-lesion times following corticofugal deprivation.

## Methods

This study was carried out in strict accordance with the recommendations in the Guide for the Care and Use of Laboratory Animals of of the Spanish Councill (Royal Decree 53/2013 - Law 32/2007) and the European Union (Directive 2010/63/EU) for the Use of Animals in Neuroscience Research. The protocol was approved by the Committee on the Ethics of Animal Experiments of the University of Salamanca (Permit Number: 2012-265). All surgery was performed under monitored anesthesia, and all efforts were made to minimize suffering. Forty-two young male Wistar rats weighing between 250–300 g were used in this study. According to the experimental procedure, animals were divided into 4 experimental groups (n = 7), as follows:

Group 1 - Animals bilaterally ablated and ABR recording prior to surgery and at PSD1 and PSD7.

Group 2 - Animals bilaterally ablated and ABR recording prior to surgery and at PSD15 and PSD30.

Group 3 - Animals unilaterally ablated and ABR recording prior to surgery and at PSD1 and PSD7.

Group 4 - Animals unilaterally ablated and ABR recording prior to surgery and at PSD15 and PSD30.

It has been shown that multiple doses of anesthesia could have potential negative effects on ABR recordings, such as tolerance and dose-dependent effect over the ABR parameters [17], [18], [19]. To avoid the negative effects, the two experimental groups (PSD1–7 and PSD15–30) were used instead of repeated measurements from single animals.

Additionally, non-ablated control groups (n = 7) were tested towards assessing the effect of anesthesia on ABRs, following a temporal sequence similar to the ablated groups. The control recordings were performed 1 and 7 days after the first ABR recording (at 250–300 g) in Group 5 and 15 and 30 days later in Group 6. The analysis of these groups showed no statistically significant changes (p>0.05, df = 3, F = 4.293).

### Surgical procedure

The AC ablations were performed under anesthesia using a mixture of ketamine chlorhydrate (30 mg/kg Imalgene 1000, Rhone Méreuse, Lyon, France) and xylazine chlorhydrate (5 mg/Kg, Rompun, Bayer, Leverkusen, Germany) for anesthesia. Animals were placed in a stereotaxic frame (#900, David Kopf Ins., Tujinga, CA, EEUU) and the left superficial area of the cranial surface was surgically exposed. A window including the primary and secondary AC areas was opened in the skull, following the stereotaxic coordinates of Paxinos and Watson [20], and the AC was removed by gentle aspiration. The animals were returned to their cages after the ablations, carefully monitoring the post-surgery recovery.

### ABR recording

ABR recordings were performed using a close-field real-time signal processing system (Tucker-Davis Technologies [TDT], System 3, Alachua, Fl, USA). The system output was calibrated before the recordings using a one-quarter inch microphone (Brüel and Kjaer) to confirm that a twofold voltage increase correspond to increases in 6 dB SPL of intensity, strictly up to a maximal value of 100 dB SPL. The ABR recordings never exceeded this value.

ABR recordings were performed before and 1, 7, 15 and 30 days after the surgery (as indicated above). Animals were anesthetized and placed in a stereotaxic frame using two hollow methacrylate bars to perform the recordings. Three subcutaneous needle electrodes placed at the vertex (reference electrode), the mastoid ipsilateral to the stimulated ear (active electrode) and the mastoid contralateral to the stimulated ear (ground electrode), were used for the recordings. Stimuli consisted of a 5 miliseconds (msec) window with 1 - msec pre-stimuli period and a 0.1-msec alternating polarity click with a repetition rate of 11 bursts/s, delivered in 10-dB ascending steps from 10 to 90 dB (Sound Pressure Level, SPL). The stimuli were delivered using a magnetic speaker through tubal earphones inserted into the external auditory channel of a single ear via the methacrylate bar. A 24-cm-long tube resulted in a 0.75-msec air conduction time of stimulus arrival at the tympanic membrane. This delay was added to the 1 msec pre-stimuli period to calculate the onset of ABR (1.7 msec). Responses were averaged 1000 times. Evoked potentials were amplified and digitized using a Medusa RA16PA preamplifier and RA4LI headstage. The final signal was filtered with a 500-Hz high-pass filter and a 3000-Hz low-pass filter.

### ABR Analysis

The ABR magnitude was measured in terms of the mean peak voltage during the 5-msec response window immediately before the stimulus onset. The quality of each recording was assessed measuring the mean background voltage of the 1-msec period before the stimulus onset. The ABR threshold was defined as the stimulus level that evoked a mean voltage +2SD above the mean background activity.

**Figure 1:**
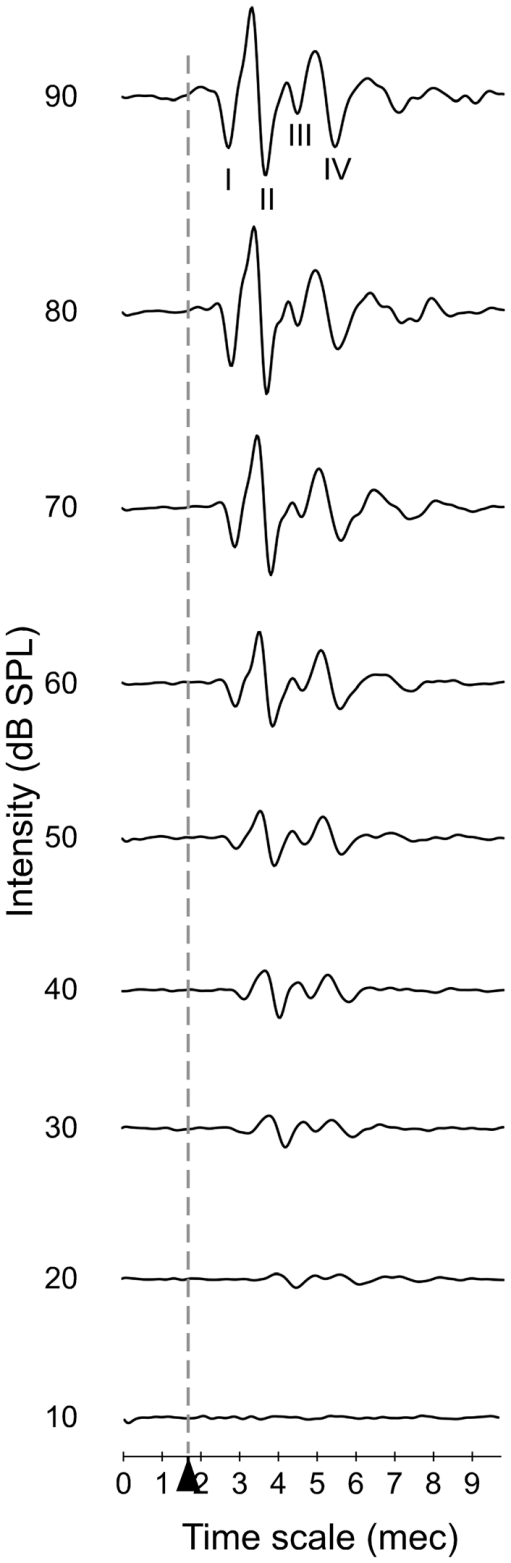
An example of the ABR waveforms obtained from a control animal during recordings from 10 to 90(msec). The stimulus onset starts at 1.7(Alvarado et al. 2012), wave II is the largest wave in rat ABR recordings whereas wave III is the smallest.

The positive and negative peaks of each wave and its latency values at 80 dB SPL were assessed using the peak analyzer application of OriginPro 8.5.1 (OriginLab, Northampton, MA) software. Wave amplitude was defined as the sum of the positive and negative peak values of each wave. The absolute wave I latency was defined as the time in msec from the onset to the positive peak of the wave. The interpeak latencies were defined as the time in msec between the positive peaks of the different ABRs waves. Statistical analyses were performed using the SPSS-IBM software, version 20 (SPSS Inc., Chicago, IL, USA). All quantitative data (amplitude and latency values) were expressed as mean value ± standard error of the mean (SEM). The differences between groups were analyzed by analysis of variance (ANOVA), using the Levene Test for analysis of homogeneity of variance, followed by the Fisher's Least Significant Difference (LSD) test for post hoc comparison, when appropriate, or the Greenhouse-Geisser test (no homogeneity of variances) and Bonferroni-test (two by two comparisons). Differences were considered statistically significant if p<0.05. Wave V was excluded from the study because it was not found in any ABR recording. The amplitude and latency percentage of change [21] was calculated using the formula: [(post-lesion value – pre-lesion value)/pre-lesion value]×100. An example of a control animal ABRs recording is showed in Fig. 1.

### Histological procedures: immunocytochemistry and lesion localization

#### Fixation and sectioning

Once the recordings protocols were completed, animals were deeply anesthetized with an intraperitoneal injection of 6% sodium pentobarbitone (60 mg/kg b.w). After a deep anesthesia, 250 ml of Ringer buffer (pH 6.9, 36°C) with 0.01% heparin were perfused through the exposed heart, followed by 1 litre of fixative, consisting of buffered 4% p-formaldehyde.

After fixation, the brains were dissected out, sectioned, and post-fixed by immersion in the same fixative solution for 2 hours before being cryoprotected by immersion in 30% sucrose in 0.1 M phosphate buffer (PB), pH 7.4, at 4°C for 48 hours. The brains were then serially sectioned in the coronal plane at 40 µm with a sliding freezing microtome.

### Immunocytochemistry for Glutamic Acid Decarboxilase and Parvalbumin

Floating sections were sequentially washed with 0.05 M TBS, pH 7.6, followed by inhibition of endogenous peroxidase by incubating with 10% methanol + H_2_O_2_ 3% in 0.1 M PB for 10 minutes. Sections were then washed in TBS-Tx, pH 7.6, for 3×15 minutes under continuous shaking. Nonspecific labelling was blocked with fetal calf serum (10%) for Parvalbumin (Pv) and normal horse serum (3%) for GAD. Sections were incubated with the corresponding primary antiserum, against Rabbit glutamate decarboxylase GAD-67; Chemicon AB108 dilution 1∶1000 in TBS-Tx (Chemicon/Millipore, Billerica, MA, USA), and Rabbit polyclonal anti Parvalbumin Swant PV28 dilution 1∶5000 in TBS-Tx (Swant, Switzerland), for 48 hours at 4°C. This was followed by washing three times in TBS-Tx for 15 minutes each. After several TBS washes, both Pv and GAD sections were incubated with an anti-rabbit biotinylated secondary antibody (biotinylated anti-rabbit IgG H + L, BA- 1000; Vector) at a dilution of 1∶200 in TBS-Tx for 120 minutes at room temperature. Subsequently, the sections were washed again in TBS-Tx and incubated in an avidin-biotinylated peroxidase complex - TBS-Tx solution for 90 minutes. The peroxidase reaction was revealed with DAB in 0.05 M Tris-HCl, pH 7.6. The control sections were not incubated with the primary antibodies to test the specificity of the detection systems.

### Cresyl violet staining

Every third serial section was stained with 1% cresyl violet (C-1791; Sigma-Aldrich), for 10 minutes. Staining differentiation was in 96% alcohol + acetic acid, and sections were finally dehydrated in graded alcohols from 50% to 100%, followed by clearing in xylene (3×3 minutes).

### Localisation of the lesions

**Figure 2:**
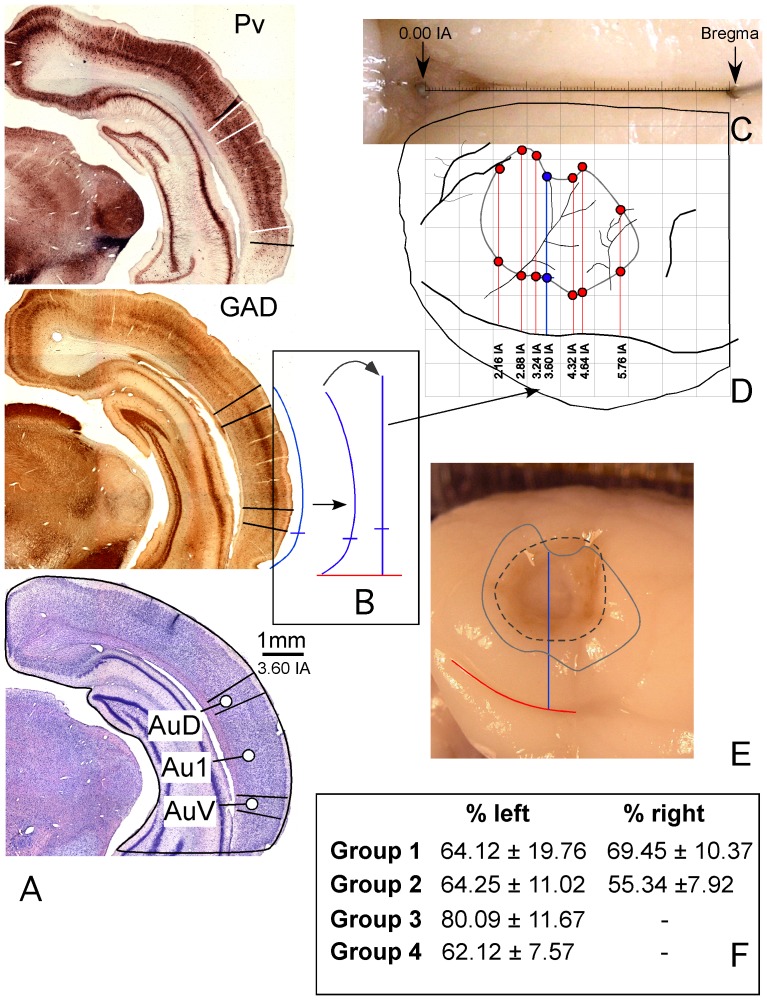
Procedure for the location and extent of lesions in the rat brain AC. **A**. Coronal caudal sections at a similar rostro-caudal level stained with Parvalbumin (Pv), Glutamic Acid Decarboxilase (GAD) and Nissl (interaural 3.60 – similar to Patxinos and Watson). In the immunostained sections, positive pyramidal cells and neuropil of layers III and V enable to define the AC boundaries (highlighted on the photographs with lines perpendicular to the surface of the section). **B**. Outline of the section contour after labeling the AC boundary. The straight line (in bold) on the right side of the curved arrow was drawn with the same length as the auditory cortex perimeter, using the Canvas software. After scaling the line to compensate the shrinkage of the section, the coordinates were transferred to the macroscopic image of the brain (thick arrow). **C**. Photograph of the brain midline with sterotactically (Patxinos and Watson coordinates) implanted needles in 0.00 IA and Bregma. **D**. AC map after transferring the coordinates of AC boundaries from seven rostrocaudal sectioning levels. **E**. Overlapping map of the cortex on a lesioned brain, positioned into a matrix to calculate the extension and location of the ablated surface relative to the AC boundaries. **F**. Results of the percentage of AC affected by lesions in the experimental groups.

The localisation of the lesions was performed as described previously [14]. Briefly, a map of coordinates was designed to localize the lesions in the ablated brains. In two control animals, a large longitudinal window was opened in the superficial area of the cranial surface from a deeply anesthetized rat and two needles were inserted into the bregma and interaural 0 references following the coordinates of Paxinos and Watson [20] (Fig. 2C). Two more needles, separated by 1 mm, were stereotactically placed in the brain to calculate the retraction of the brain with respect to the histological sections. The brain was carefully removed, after finishing the surgery, and immersed in 4% paraformaldehyde. After post-fixation, the lateral surface of the brain was photographed using a Nikon camera located 21 cm above the brain. The photograph was superimposed with a 9×9 mm grid using the Canvas X computer program to transfer the reference coordinates (Bregma and interaural 0.00) to the brain surface (Fig. 2D and E). Stained sections were photographed using a Leica DMRB microscope with ×10 objective, and mosaics were generated using a motorized stage and the Neurolucida program (MicroBright field - USA) (Fig. 2A). The limits of the AC were determined by GAD, parvalbumin, and Nissl staining in accordance with Paxinos and Watson [20]

The measurements of the distances between the limits of the AC were transferred to the brain photographs using the rhinal fissure of the brain as reference (Fig. 2D and E). This was accurately performed by compensating the error of the brain curvature using the tool “dimensioning perimeter” of the Canvas X computer software (Figs. 2 B). The resulting map (Fig. 2 D) was superimposed with photographs from the ablated brains (Fig. 2E), and the percentage of lesion was calculated relative to the area occupied by the AC.

## Results

### Localisation of lesions

**Figure 3:**
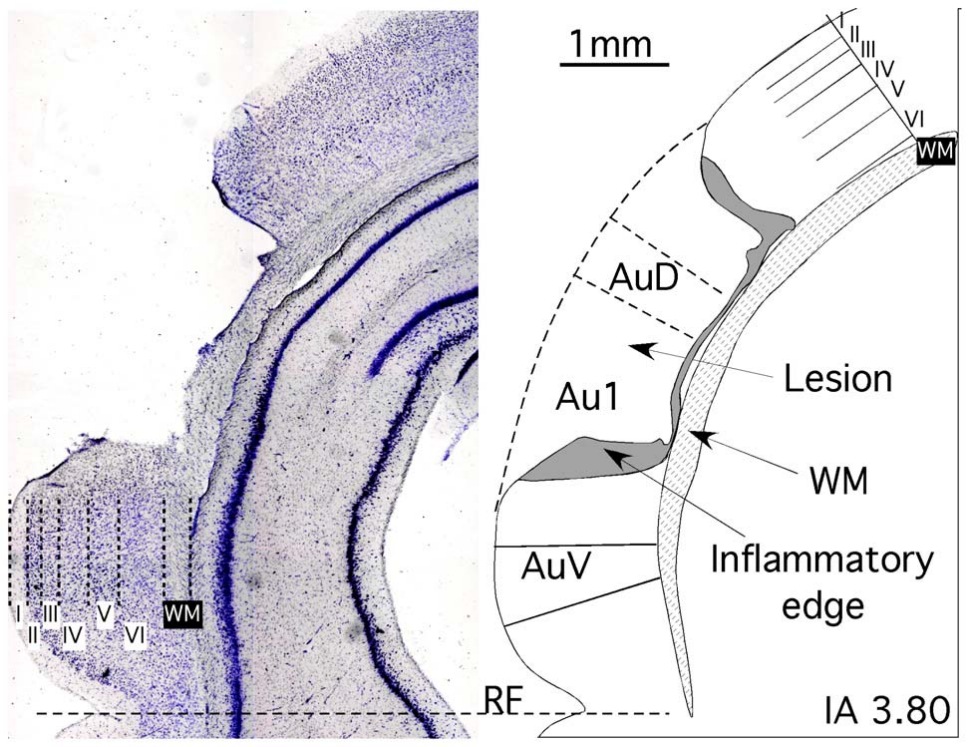
Example of section from animals received lesions. Left, a Nissl stained section showing the different cortical layers and the ablated area. **Right**, the corresponding drowing line, showing the AC subdivisions affected by the ablation. Note that lesion affect all AC layers but not the underlying white matter (WM).

The analysis of the ablated AC area showed that lesions included a region ranging from 70 to 100% of the total AC area. Fig. 2 F summarizes the mean percentage ± SEM of the lesion from each group of animals, and Figure 3 shows and example of a section from animals received a lesion.

### ABR recordings

#### Threshold

The ABR threshold of the bilateral ablated animals before surgery was 11.29±1.23 dB SPL in the short-term group (PSD1–7) and 15.97±1.49 dB SPL in the long-term group (PSD15–30). After surgery, the ABR threshold of animals from the short term group increases to 14.50±1.00 dB SPL at PSD1 and 16.93±1.73 dB SPL at PSD7, while animals from the long term group showed ABR thresholds similar to the pre-ablation condition both at PSD15 and 30 (14.70±0.95 and 14.78±0.86 dB SPL, respectively). The increase in threshold by 28.43% at PSD1 and 49.95% at PSD7 was statistically significant (p<0.05, F = 3.660, df = 2). However, changes in the ABR threshold at PSD15 and 30 were not statistically significant.

The ABR recordings of animals from the unilateral ablated groups were divided into those in which the ear ipsilateral to the lesion was used and those in which the contralateral ear to the lesion was used. When the ear ipsilateral to the lesion was used, the animals from the short-term group showed an ABR threshold before surgery of 19.72±1.23 dB SPL and the animals from the long-term group showed a threshold of 18.47±2.28 dB SPL, while the ABR thresholds after injury were 14.53±2.72 dB SPL at PSD1, 14.01±1.29 dB SPL at PSD7, 19.46±3.85 at PSD15 and 17.62±2.41 dB SPL at PSD30. Stimulation in the ear contralateral to the lesion showed an ABR threshold before the ablation of 12.61±1.73 dB SPL in the animals from the short-term group and 13.13±1.64 dB SPL in the animals from the long-term group, while the thresholds after surgery were 14.63±2.57 dB SPL at PSD1, 14.47±0.87 dB SPL at PSD7, 13.31±0.76 at PSD15 and 15.54±0.78 dB SPL at PSD30. No statistically significant differences were found in the ABR threshold of the unilateral ablated animals, at any PSD, regardless of the ear used for stimulation.

#### Wave Amplitudes

**Figure 4:**
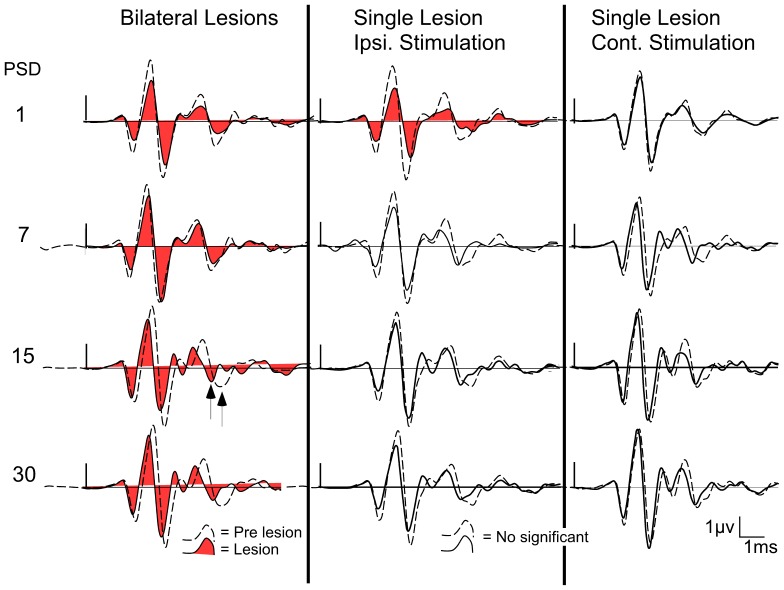
Average ABRs waveform before and after cortical ablation from all experimental groups (n = 7), at different post-surgery days (PSD): at left, ABR waveforms from animals with bilateral AC lesions; in the middle, ABR waveforms of the ear ipsilateral to the AC ablation from the unilaterally ablated animals; at right, ABR waveforms of the ear contralateral to the AC ablation from the unilaterally ablated animals.

**Figure 5:**
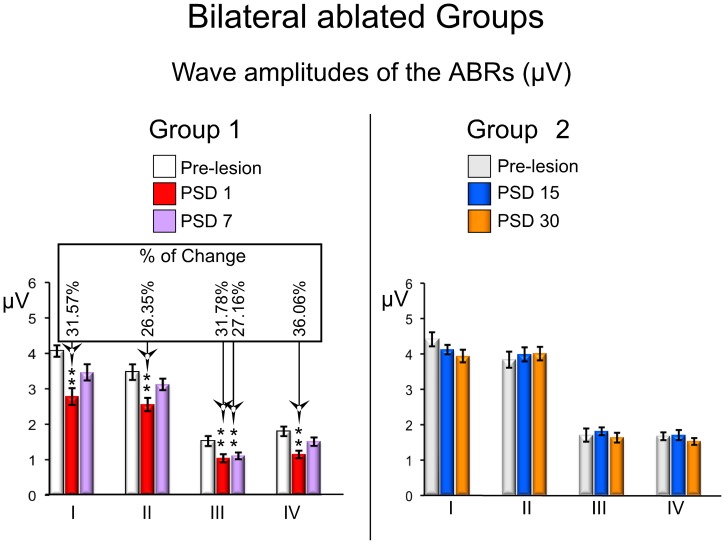
ABRs changes in waves amplitudes from the bilateral ablated experimental groups. The mean±SEM amplitude of waves in microvolts (µV) is shown in the graphs. * p<0.05. ** p<0.01. *** p<0.001. The percentage of change (Alvarado et al 2007) regarding the pre-lesion condition is shown in the top box. Note that all mean values of waves decrease in relation to the pre-lesion condition in the short-term (PSD1 and 7) periods, and no significant changes were observed at long-term (PSD 15 and 30).

The amplitudes of all ABR waves assessed (I–IV) in the short-term group of the bilateral ablated animals showed a significantly decrease in both PSD1 and PSD7 experimental animals, compared to those values before the surgery (Fig. 4 and 5). The PSD1 experimental condition showed a significant decrease in wave I by 31.57% (df. = 2; F = 13.842), in wave II by 26.35% (df. = 2; F = 9.216), in wave III by 31.78% (df. = 2; F = 21.097) and in wave IV by 36.06% (df. = 1.359; F = 14.015) (Fig. 5 left). Wave III also showed a statistically significant decrease of 27.16% at PSD7, although the decrease in the amplitude of waves I, II and IV in this experimental condition (PSD7) was not statistically significant in relation to the pre-lesion condition (Fig. 5 left). ABRs from the bilaterally ablated long-term group showed wave amplitudes after the injury similar to those in the pre-lesion condition, and no statistically significant changes were found either in PSD15 or PSD30 (Fig. 4 and Fig. 5 right).

**Figure 6:**
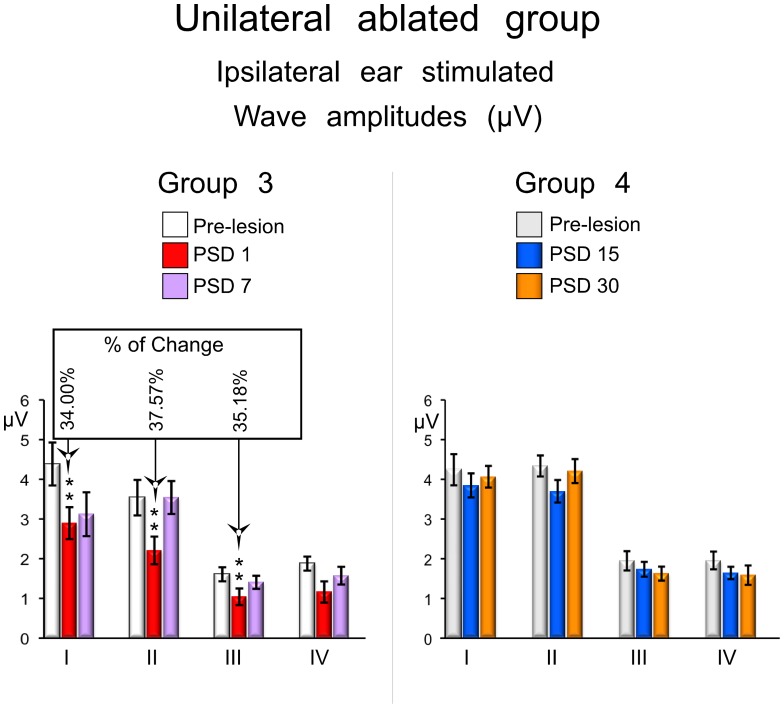
ABRs changes in waves amplitudes from the unilateral ablated experimental groups. The mean±SEM amplitude of waves in microvolts (µV) is shown in the graphs. * p<0.05. ** p<0.01. *** p<0.001. The percentage of change (Alvarado et al 2007) regarding the pre-lesion condition is shown in the top box. Note that mean values of waves I, II and III decrease in relation to the pre-lesion condition in the short-term (PSD1 and 7) periods, and no significant changes was observed at long-term (PSD 15 and 30).

The ABR recordings in the unilaterally ablated animals were divided into those in which the ear ipsilateral to the lesion was used and those in which the ear contralateral to the lesion was used for sound stimulation. Using the ear ipsilateral to the lesion, rats from the short-term group showed a decrease in wave amplitudes at PSD1 (Fig. 4 and 6). This experimental condition showed a statistically significant decrease of wave I of 34% (p<0.05; df. = 2; F = 6.759), wave II of 37.57% (p<0.01; df. = 2; F = 17.294) and wave III of 37.57% (p<0.05; df. = 2; F = 5.565) (Fig. 6 left). The decrease in the amplitude of wave IV was not statistically significant (Fig. 6 left). ABRs recorded during stimulation of the ipsilateral ear from the long-term group showed wave amplitudes after injury similar to those to the pre-lesion condition, and no statistically significant changes were found either at PSD15 or PSD30 (Fig. 4 and Fig. 6 right).

When the ear contralateral to the lesion was used, rats from both short- and long-term groups showed wave amplitudes after surgery similar to those in the pre-lesion condition, and no statistically significant changes were found at any PSD (Fig. 4).

#### Waves latencies

**Figure 7:**
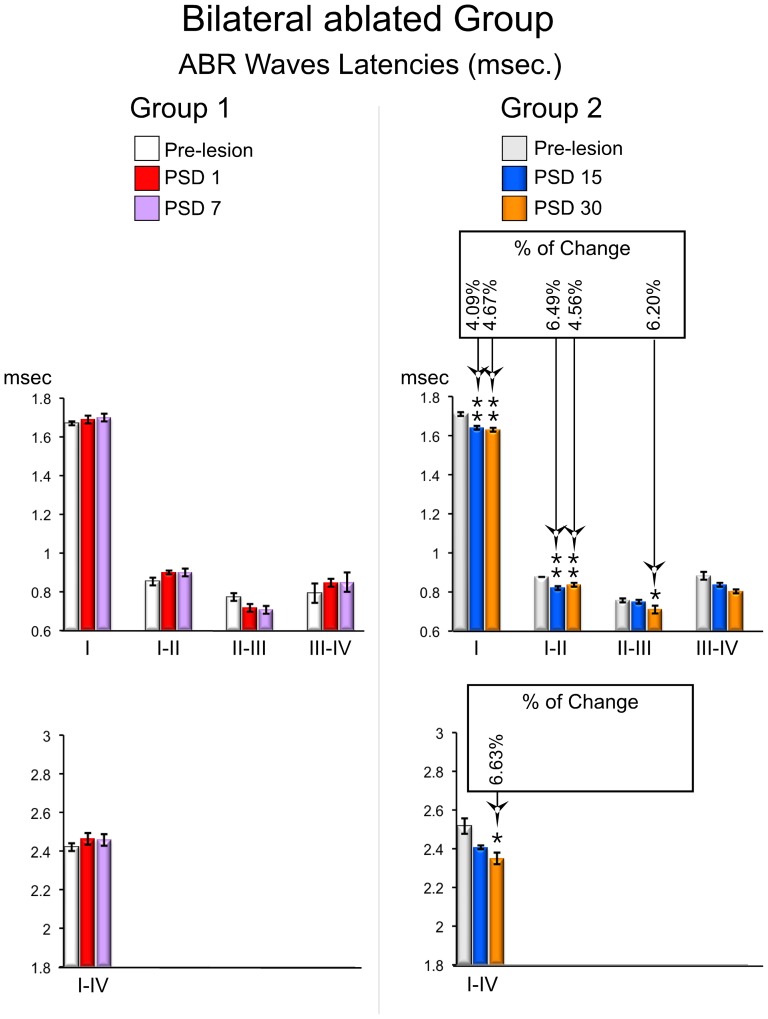
ABRs changes in waves latencies from the bilateral ablated experimental groups. The mean±SEM of significant latencies of all intervals of waves in milliseconds are show on the graphs. * p<0.05. ** p<0.01. *** p<0.001. The percentage of change (Alvarado et al 2007) regarding the pre-lesion condition is shown in the top box. Latencies are significantly shortened in recordings conducted in the long-term (PSD 15 and 30).

The ABR from the short-term group of the bilaterally ablated animals showed absolute latencies of wave I and interpeak latencies of waves after injury similar to those in the pre-lesion condition, and no statistically significant changes were found either in PSD1 or PSD7 (Fig. 4 and Fig. 7 left). However, there was a shortening in the latencies of the long-term (PSD15 and 30) animals (Fig. 4 and Fig. 7 right) This experimental condition showed a statistically significant shortening in the absolute latency of wave I of 4.09% at PSD15 and 4.67% at PSD 30 (p<0.01; df. = 2; F = 10.458), and a statistically significant shortening in the interpeak latency of waves I–II of 6.49% at PSD15 and 4.56% at PSD30 (p<0.05; df = 2; F = 8.100) (Fig. 7 right). Furthermore, the interpeak latencies of waves II–III and I–IV were shortened at PSD30 by 6.20% (p<0.05; df. = 2; F = 4.683) and 6.63% (p<0.05; df. = 2; F = 10.81), respectively (Fig. 7 right). The interpeak latency of waves III–IV did not show any statistically significant change at PSD 15 and 30 (Fig. 7 right).

**Figure 8:**
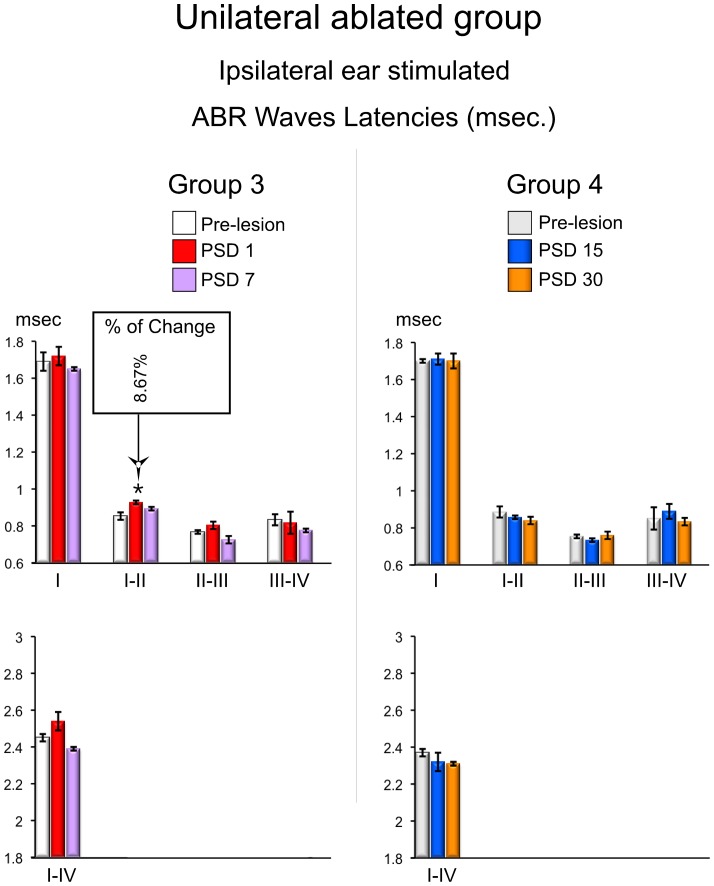
ABRs changes in waves latencies from the unilateral ablated experimental groups. The mean±SEM of significant latencies of all intervals of waves in milliseconds are show on the graphs. * p<0.05. ** p<0.01. *** p<0.001. The percentage of change (Alvarado et al 2007) regarding the pre-lesion condition is shown in the top box. Latencies of wave I–II are significantly increased in recordings conducted in the sort-term (PSD 1).

The ABR recording in the unilaterally ablated animals were divided according to the ear used to deliver the sound stimulation into those in which the ear ipsilateral to the lesion was used and those in which the ear contralateral to the lesion was used, as previously indicated. Animals from both short- and long-term groups showed ABR latencies after injury during the stimulation of the ear ipsilateral to the lesion similar to those in the pre-lesion condition, except the interpeak latency of waves I–II, which were significantly increased by 8.67% (p<0.05; df. = 2; F = 4.252) at PSD1 (Fig. 4 and 8).

Rats from both short- and long-term groups showed wave latencies after surgery similar to those in the pre-lesion condition when the ear contralateral to the lesion was used, and no statistically significant changes were found at any PSD (Fig. 4).

## Discussion

Changes in auditory brainstem evoked responses found in the present study depended on the type of lesion (single or bilateral) induced in the experimental animals and the post-surgery time of ABR recordings. Accordingly, bilateral AC ablations caused a significant increase in the thresholds of ABR recordings at PSD1 and 7. Conversely, unilateral AC ablation did not have any statistically significant effect on the auditory thresholds at any PSD. The wave amplitudes significantly decreased at PSD1 and 7 following bilateral AC ablations, while a decrease in wave amplitude only occurred following unilateral AC ablations at PSD1, and then only upon stimulation of the ear ipsilateral to the lesion. Finally, no effects on the latencies were noted in the bilaterally ablated group at PSD1 and 7. However, statistically significant decreases in latency were found at PSD15 and 30. A statistically significant increase in wave I–II interpeak latency was found at PSD1 following unilateral ablation, albeit just upon stimulation of the ear ipsilateral to the lesion, as previously indicated (Table 1 summarizes all these results).

**Table 1:** Summary of the changes in the ABR statistical analysis in the different experimental groups.

Bilateral lesions					Unilateral lesion Ipsilateral stimulation				Unilateral lesion Contralateral stimulation			
PSD	1	7	15	30	1	7	15	30	1	7	15	30
Threshold	↑*	↑*	–	–	–	–	–	–	–	–	–	–
Amplitudes												
W. I	↓**	–	–	–	↓*	–	–	–	–	–	–	–
W. II	↓**	–	–	–	↓**	–	–	–	–	–	–	–
W. III	↓**	↓**	–	–	↓*	–	–	–	–	–	–	–
W. IV	↓**	–	–	–	–	–	–	–	–	–	–	–
Latencies												
W. I	–	–	↓**	↓**	–	–	–	–	–	–	–	–
Ws. I–II	–	–	↓*	↓*	↑*	–	–	–	–	–	–	–
Ws. II–III	–	–	–	↓*	–	–	–	–	–	–	–	–
Ws. III–IV	–	–	–	–	–	–	–	–	–	–	–	–
Ws. I–IV	–	–	–	↓**	–	–	–	–	–	–	–	–

W(s) = wave(s). On = onset.

* p<0.05.

** p<0.01.

*** p<0.001.

The ABR threshold is the lowest intensity (in dB SPL) that evokes a peak voltage response corresponding to a minimum of 2SD above the mean background activity. Wave II is the largest wave in rat ABR recordings [22] and it is frequently used to calculate the threshold value. The ABR threshold could be defined as the lowest intensity at which an electrically evoked response is produced by the auditory brainstem because the waves in ABR recordings of these animals are generally accepted to correspond to the activation of the auditory nerve, cochlear nuclei, superior olivary complex and lateral lemniscus (waves I, II, III and IV, respectively) [23]. It has been previously shown that this electrical activity evoked in the brainstem could be attenuated or eliminated by lesions in the auditory nerve [23], [24]. This has lead to suggesting that the increase in ABR threshold and decrease in wave amplitude following bilateral AC ablations, reported herein, could be explained as a consequence of the decrease in activity of the organ of Corti and the auditory nerve, upon removal of cortical inputs. In the cochlea, depolarization and firing of spiral ganglion type I neurons may be regulated in two ways, by modulating the glutamatergic activation of inner hair cells (IHC), through LOC activation, and/or indirectly by changes in the micromechanical activity of the outer hair cells (OHC), through MOC activation [25]. The LOC efferent system may alter the resting potential or “set-point” of distal dendrites of type I spiral ganglion neurons, changing its sensitivity to IHC glutamatergic activation [25]. The activity of MOC fibres hyperpolarizes OHC and adjusts its axial stiffness, thereby modulating its motility and changing the gain of the cochlear amplifier [26], [27], [28], [29]. Previous studies have shown an effective cortical modulation in the activation of type I spiral ganglion distal dendrites. Accordingly, decreases in compound action potential amplitudes after cooling or lidocaine inactivation of the AC [11] suggest a top-down control of the auditory nerve fibres. Furthermore, changes in cochlear microphonic amplitudes and otoacoustic emissions have been shown following AC electrical stimulation [7], [8] and AC resection or deactivation by muscimol [7], [30] indicating that AC may regulate OHC activity.

Considering the combined effect of the descending projections on the medial and lateral efferent systems, we suggest that the changes in threshold and waves amplitude noted in our experiments following bilateral AC ablation could be explained by the loss of descending inputs on MOC and/or LOC neurons. This loss could induce a decrease in the activity of these systems and, consequently, reduce their effect on the cochlea. Anatomical studies using retrograde and anterograde tracing methods also support this hypothesis. For example, close contacts have been shown between MOC neurons and corticofugal terminals in the VNTB [4]. Furthermore, terminal fields from the AC have been identified in the LSO and dorsal periolivary region, where LOC neurons are located [3]. In addition, the increased thresholds and decreased wave amplitudes following bilateral cortical ablation could also suggest a reinforcing AC role on the excitation of the caudal auditory pathway because the corticofugal projection seems to have an excitatory and potentially glutamatergic role [6]. Therefore, the findings in the present study support the hypothesis that inputs from the AC could act as drivers of the lower brainstem auditory nuclei and their removal would impact the activity of those nuclei.

Both threshold values and wave amplitudes reverted to control levels at the longest post-surgery periods, that is, PSD15 and 30. Our cortical lesion may cause the degeneration of fibres directly connecting the cortex to the olivo-cochlear neurons and to those connecting to the midbrain. Previous studies from our laboratory have shown that cortical ablation produces a decrease in c-Fos immunoreactivity in the IC, which is restored in long post-surgery periods in experimental animals [15]. This long-term reactivation of the IC should also affect colliculo-olivary connections and, therefore, could be involved in the recovery (PSD15 and 30) of ABR wave parameters found in the present study, because the electrical stimulation of the IC has shown to modulate the cochlear afferent response by the olivo-cochlear system [31]. Further studies assessing the electrical stimulation of the IC and performing electrophysiological recordings in the superior olivary complex in animal models, are required to confirm this hypothesis.

Moreover, a long-term functional adaptation of inner ear hair cell synapses should also be considered. Altering the balance of excitation and inhibition in a central nervous system (CNS) network affects the synthesis and trafficking of postsynaptic receptors and channels (scaling), thereby modifying the synaptic strength and conductance of axons [32]. These changes lead to a dynamic self-regulating process known as homeostatic plasticity, which is maintained until the network recovers its balance (set point level) [33]. A synaptic scaling mechanism has been suggested in the cochlear nuclei as a response to changes in auditory nerve activity following unilateral occlusion of the external ear canal in rats by unilateral ear plugging [34]. These authors show that globular cells, intermediaries in the chain that forms the cochlear-brainstem feedback loop, up-regulate the synthesis of the α-amino-3-hydroxy-5-methyl-4-isoxazolepropionic acid (AMPA) receptor subunit 3 (GluA3; excitation) and down-regulates glycine receptor alpha 1 subunit (GlyRalpha1; inhibition), as a compensatory homeostatic response to decreased activity in the auditory nerve.

A synaptic scaling homeostatic mechanisms to rebalance the olivo-cochlear system in the inner hair cells and superior olivary complex or cochlear nuclei may explain the recovery of ABRs shown after PSD7. Ongoing studies, using qPCR analysis of several genes in the organ of Corti after AC lesion of our laboratory appear to support this hypothesis (data not shown).

The correlation between waves in ABR recordings and auditory nuclei, and the tracts which generated them, may be understood based on studies assessing small lesions in the caudal auditory pathway of cats [35], [36], [37]. Additionally, a similar approach using radiofrequency lesions performed in rats has shown changes in wave III of ABR recordings after altering the superior olivary complex or lateral lemniscus [23]. Interestingly, the present study has also shown that wave amplitudes were restored at PSD 7, except for wave III (Fig. 3 bottom left). This finding suggest a more complicated mechanism of recovery of electrical activity in the superior olivary complex, and most likely reflects a direct effect of the lost of excitation resulting from the removal of the descending corticofugal terminals.

A significant shortening in latency was noted in recordings after long post-surgery periods (PSD15 and 30) following bilateral cortical lesions. The reduction in latency time could be explained by the theory of cochlear filtering, which postulates that a wider frequency tuning produces a shortening of responses. A shortening of response leads to activate faster nerve responses and consequently also the latencies of ABR waves. Therefore, afferent neurons may trigger earlier if the cochlear filtering is reduced by MOC alterations or OHC injury (as reviewed by [38]. Other experimental evidences also support the notion that changes in MOC activity may generate a decrease in response latency [39], [40], [41], [42], [43]. Even clinical results also appear to support this hypothesis by showing that a broader frequency tuning, as a result of peripheral hearing loss, causes the decreased latencies found in ABR recordings of deaf patients [44]. As the above studies appear to indicate, the shortening of latencies noted in the present study at PSD15 or 30, may be explained by an imbalance in cochlear filtering resulting from the long-term reorganization of inputs in MOC neurons following AC deprivation. Moreover, we cannot exclude changes in processing in caudal brainstem nuclei, which may also explain this finding. The hypothesis of a scaling mechanism involved in the long-term functional recovery of auditory thresholds and wave amplitudes in the bilaterally ablated animals imply an enhancement that may affect the latencies of auditory responses. The increased strength towards recovering the auditory system “normal” function could also affect other aspects of the neuronal responses, including the reaction time. Consequently, neuronal latencies could be accelerated. Therefore, the shortening of latencies could be explained by a combination of cochlear filtering and central synaptic scaling.

The descending projection to the auditory brainstem ends bilaterally but with a weak contralateral component [3], [5], [13], whereby a different effect may be expected in single cortical ablation experiments, when stimulating the ear ipsilateral or contralateral to the lesion. Accordingly, we have found that ipsilateral ear stimulation one day after unilateral ablation, shows statistically significant changes in the amplitude of waves I through III and an increase in the latency between waves I and II. Nonetheless, no significant changes have been noted upon contralateral ear stimulation at any PSD following the unilateral cortical removal. Previous studies from our laboratory have shown that unilateral AC ablations increase the ABR thresholds at PSD15 [45]. This discrepancy, could be explained by differences in the size and location of lesions in the cortex and the fact that different anesthetic combinations were used in each study. Previous studies [19], [46] have shown that the ketamine and medetomidine, used in the study of Clarkson et al. [45] may have a negative effect on the evoked potentials.

The results reported herein suggest that both changes in evoked electrical activity and their reversal over time depend on the number of connections lost after AC lesions and reinforce the hypothesis that the descending control acts as an enhancer of the auditory response.
