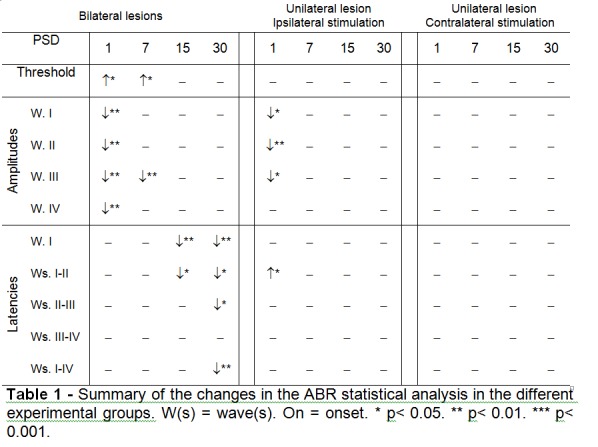# Correction: Long-Term Evolution of Brainstem Electrical Evoked Responses to Sound after Restricted Ablation of the Auditory Cortex

**DOI:** 10.1371/annotation/07ec3908-23dd-4c31-926d-9d73a2815032

**Published:** 2013-11-12

**Authors:** Verónica Lamas, Juan C. Alvarado, Juan Carro, Miguel A. Merchán

The affiliations for Miguel Merchan and Juan Carro are incorrect. They are affiliated with 1, the Instituto de Neurociencias de Castilla y León, Universidad de Salamanca, Salamanca, Spain. Please see the full affiliation below:

Verónica Lamas 1, Juan C. Alvarado 2, Juan Carro 1, Miguel A. Merchán 1

1. Instituto de Neurociencias de Castilla y León, Universidad de Salamanca,

Salamanca, Spain

2. Instituto de Investigación en Discapacidades Neurológicas (IDINE),

Facultad de Medicina de Albacete, Universidad de castilla-La Mancha,

Campus in Albacete, Spain

The column headings in Table 1 are not appropriately aligned. Please see the corrected Table 1 here: 

**Figure:**